# Rethinking the terminology: transitioning from ‘junior doctors’ to ‘postgraduate doctors’ for enhanced representation in modern medical practice

**DOI:** 10.3399/BJGPO.2023.0178

**Published:** 2023-11-29

**Authors:** Sai Ram Pillarisetti, Chandra Kanneganti, Azeem Majeed, Kaveh Asanati

**Affiliations:** 1 British International Doctors Association, Stockport, UK; 2 University Hospitals North Midlands NHS Foundation Trust, Stafford, UK; 3 Institute of Medicine, University of Bolton, Bolton, Greater Manchester, United Kingdom; 4 Department of Primary Care and Public Health, Imperial College London, London, UK; 5 National Institute for Health Research Applied Research Collaboration Northwest London, Imperial College, London, UK

**Keywords:** junior doctor, Postgraduate education, postgraduate doctor, resident doctors

In the ever-evolving landscape of modern-day health care, re-evaluating the language used to describe medical professionals is essential. The term ‘junior doctor’ has come under increased scrutiny^1^ because of its pejorative connotations, prompting the British Medical Association (BMA) to call for its revision to accurately reflect the expertise and responsibilities of these professionals.^1^

Traditionally, ‘junior doctor’ referred to doctors in training or those in their early postgraduate years. Its origin remains unclear, but it has been used in the UK for decades to encompass doctors below the level of a consultant.^2^ Hence, the term encompasses doctors who may be in the very early stages of their postgraduate medical training through to doctors who may have 10 years or more training. However, as the role of these doctors has evolved, so too should the terminology we use to describe them.

While gaining traction during the 2016 junior doctor strikes, the term ‘junior doctor’ falls short of capturing the knowledge and experience gained through medical education and training.^3^ It may imply that these doctors are inexperienced and incompetent; this is not accurate as they are fully qualified medical practitioners who play a crucial role in patient care. They are responsible for a wide range of duties, including diagnosing and treating patients, performing interventional procedures, and making important medical decisions. The term may also diminish the value and importance of the work that junior doctors do.

As medicine advances and doctors’ roles expand, updating terminology is crucial to align with contemporary realities. It can affect how patients, colleagues, NHS managers, politicians, and the public perceive these professionals. To address this, adopting a more accurate and respectful label, such as ‘postgraduate doctors’ may better represent their level of education and training.

This is an important issue for primary care as well as for hospital medicine. All GPs will have spent several years in traditional ‘junior doctor’ roles and may have experienced instances where they felt the term ‘junior’ was not reflective of the skills they employed to fulfil their responsibilities as critical members of the clinical team.

Equally, many GPs act as supervisors to these doctors in clinical practice — such as GP registrars and foundation doctors — and keeping up to date with terminology is important as it nurtures a shared feeling of mutual respect between trainer and trainee. This, in turn, improves job satisfaction and ultimately influences recruitment into medical specialties, including general practice. At a time when recruitment into general practice roles is a major challenge for the UK’s NHS and for many health systems in other countries, it is more important than ever to recognise perspectives of the younger generation of doctors.

A recent independent report by Prof Scarlett McNally for Health Education England (HEE) found that 88% of almost 2000 respondents, including doctors, patients, and healthcare staff, deemed the term ‘junior doctor’ inappropriate due to its vagueness and lack of representation.^4^ Additionally, 60% believed the term should be avoided altogether. ‘Postgraduate doctors’ was the most accepted alternative (60%).^4^ Moreover, 90.7% of respondents wanted to know the seniority level if the doctor is not a consultant.^4^ For instance, referring to doctors based on titles like ‘FY1 doctor in general surgery’ or ‘academic clinical fellow (ACF) in general practice’ acknowledges their roles and specialisation.

Modern health care involves doctors at all stages of training taking on significant responsibilities including in general practice where foundation doctors and GP registrars are important members of the primary care team. The term ‘junior doctor’ fails to acknowledge their vital roles in patient care, decisionmaking, and clinical leadership. Embracing a revised terminology that recognises them as ‘postgraduate doctors’ ensures that their expertise and contributions are better acknowledged and valued by patients in all healthcare settings, including primary care.

The landscape of medical careers has changed in recent years. In 2011, 71% of doctors chose to enter specialty training immediately after Foundation Year 2 (F2), now that figure has dropped to 35%.^5^ As a result, many doctors with substantial postgraduate experience are now occupying roles traditionally held by ‘junior doctors’.^6^ This shift, coupled with ‘imposter syndrome’ phenomenon — where these doctors may feel inadequate despite their qualifications — and the potential for being mistaken as newly qualified doctors, can create challenges in delivering optimal patient care. Therefore, updating the terminology to better reflect their experience and qualifications could have a positive impact on their professional development and autonomy.^4^

To implement this change effectively, doctors should introduce themselves to patients and colleagues as a ‘doctor’ or ‘surgeon’ and, when necessary, as ‘postgraduate doctors’ or their specific grade, such as ‘ST4 doctor’ or ‘GP registrar’. Clear designations on name badges reduce uncertainty and potential unconscious bias within medicine.^4,7^

Furthermore, patients may be unfamiliar with specific grades, especially if they are being reviewed in the middle of the night or at times of acute distress. Introducing oneself as a ‘junior doctor’ may inadvertently cause further anxiety and apprehension. A shift towards introducing oneself as a ‘doctor’ or ‘postgraduate doctor’ instils more confidence and highlights the expertise and capabilities of the medical professional.

Some surgical and medical royal colleges in the UK have also expressed concern over the use of the term ‘junior doctors’ and have shown support for a shift away from this term, as reflected in their statements over the years.^4^ This collective effort from the medical community emphasises the significance of re-evaluating language in health care.

The language used to describe healthcare professionals significantly impacts patient perception and trust.^7^ A more accurate and descriptive term helps patients better understand the qualifications and experience of the doctors providing their care. This fosters trust and confidence in the healthcare system, as patients can better appreciate the expertise and level of training possessed by these medical professionals.

As health care continues to evolve, re-examining the language used to describe medical professionals is crucial. The term ‘junior doctor’ no longer adequately reflects the skills, knowledge, and responsibilities of doctors in training or in their early postgraduate years.^1^ By revising this title and adopting the term ‘postgraduate doctors’, while using specific titles based on individual roles, we can create a more accurate, respectful, and inclusive terminology. This change not only empowers these professionals but also has the potential to enhance patient care. The medical community should engage in discussions and work towards implementing a revised designation that better represents the realities of modern medical practice.

## Data

N/A
